# B cell-mediated immune reconstitution after lung transplantation: mechanisms, interventions, and prognostic evaluation

**DOI:** 10.3389/fimmu.2026.1798139

**Published:** 2026-06-24

**Authors:** Yaru Liu, Licheng Song, Ruofan Su, Keruo Wang, Dingyun Song, Yi Yang, Junjie Du, Bo Wu, Lixin Xie

**Affiliations:** 1 Senior Department of Pulmonary and Critical Care Medicine, the Eighth Medical Center of Chinese PLA General Hospital, Beijing, China; 2 Department of Urology, Tianjin Institute of Urology, The Second Hospital of Tianjin Medical University, Tianjin, China; 3 Lung Transplant Center, Wuxi People’s Hospital Affiliated to Nanjing Medical University, Wuxi, China

**Keywords:** AMR, B cells, BCR repertoire, DSA, immune monitoring, lung transplantation

## Abstract

Lung transplantation remains a life-saving option for patients with end-stage lung disease, yet long-term outcomes remain limited by chronic rejection and infection. Although T cells have historically been the focus of transplant immunology, B cells are now recognized as critical regulators of immune responses through the production of antibodies, antigen presentation, and cytokine secretion. These functions contribute to both protective immunity and antibody-mediated rejection (AMR), particularly in the form of donor-specific antibodies (DSAs), which drive chronic lung allograft dysfunction (CLAD). B cell dysregulation can result from ischemia-reperfusion injury (IRI), infection, and immunosuppressive agents such as tacrolimus (TAC), with distinct effects emerging across perioperative and late post-transplant phases. Advances in single-cell RNA sequencing and B cell receptor (BCR) repertoire profiling have enabled precise characterization of B cell dynamics, offering new opportunities for stratified immune monitoring and risk prediction. This review summarizes B cell biology in lung transplantation, outlines stage-specific perturbations, and explores how multi-omics technologies may support personalized immunomodulation and prognostic assessment.

## Introduction

Organ transplantation remains a cornerstone therapy for patients with end-stage organ failure, offering significant improvements in both survival and quality of life (1). Despite reduced rates of acute rejection due to improved immunosuppressive strategies (2–4), long-term outcomes remain suboptimal due to chronic rejection and infection. While immunosuppressive therapies are essential for preventing immune-mediated graft damage, they also impair the host’s defense mechanisms, rendering recipients highly vulnerable to opportunistic infections—now a leading cause of post-transplant mortality (5, 6).

Lung transplantation presents the greatest immunologic burden among solid organ transplants, with a 5-year survival rate of ~55%—notably lower than that of kidney or liver transplantation (2). This vulnerability is due to continuous exposure to environmental antigens and susceptibility to IRI, complement activation, and innate immune overactivation (7).

While T cell–mediated rejection has traditionally been the primary focus in transplant immunology, emerging evidence underscores the central role of B cells in orchestrating both protective and pathological immune responses. Beyond their established contribution to antibody-mediated rejection (AMR) through the production of donor-specific antibodies (DSAs), B cells also modulate T cell responses via antigen presentation and cytokine secretion (8). These multifaceted roles position B cells as central players in post-transplant immune dysregulation, with subset imbalances linked to increased infection risk and graft dysfunction. However, agents such as TAC (9), while effective in suppressing T cell activation, may cause excessive immunosuppression, leading to B cell dysregulation—manifested as memory B cell expansion, aberrant activation, and plasmablast accumulation (10).

In this review, we synthesize the current understanding of B cell–mediated immunity following lung transplantation. We discuss the roles of B cells in rejection and host defense, examine how ischemia-reperfusion injury (IRI), infection, and immunosuppressive agents such as tacrolimus shape B cell responses, and explore the potential of multi-omics platforms for immune monitoring and prognostic assessment across post-transplant phases.

## 1 Immunologic mechanisms and postoperative complications following lung transplantation

### 1.1 Inherent susceptibility of lung transplants and postoperative complications

Lung transplantation presents unique immunological challenges due to the lung’s continuous exposure to the external environment (11). As the only solid organ with an open mucosal barrier and specialized immunoregulatory features, the lung allograft is particularly vulnerable to microbial invasion and inflammatory injury after transplantation (12). Clinically, common pathogens such as Pseudomonas aeruginosa, CMV, EBV, and Aspergillus contribute substantially to morbidity and mortality (13). Moreover, perioperative factors, including airway manipulation and ischemia–reperfusion injury, can disrupt epithelial integrity, impair barrier function and mucociliary clearance, and thereby facilitate antigen entry and local immune sensing, creating conditions for the interplay between infection and rejection (14, 15).

Unlike relatively sterile transplanted organs such as the kidney, heart, and liver, immune responses in the lung rely more heavily on compartmentalized regulation at the airway–alveolar interface and on barrier coordination. Under homeostatic conditions, alveolar macrophages and epithelial cells together form a local regulatory network that helps prevent chronic immune activation despite continuous exposure to inhaled antigens (16).

Notably, GM-CSF derived from alveolar type 2 (AT2) cells is critical for maintaining alveolar macrophage identity, while TGF-β cooperates with GM-CSF to establish their tissue-specific program (17, 18). Airway epithelial cells may also actively shape local humoral immunity by expressing BAFF and APRIL and further upregulating them upon stimulation, thereby supporting local B cell survival, differentiation, and immunoglobulin responses (19, 20). However, donor-derived resident immune cells in the graft are gradually replaced by recipient-derived cells, a process that reshapes the local immune ecosystem and may disturb innate immune reconstitution, thereby further increasing immunologic vulnerability (21).

Within this mucosal immune environment, mucosa-associated lymphoid tissue (MALT), particularly bronchus-associated lymphoid tissue (BALT) and inducible BALT (iBALT), may develop in response to chronic inflammation, infection, or tissue injury, providing a local niche for antigen presentation and T- and B cells interactions that is not entirely dependent on draining lymph nodes. In lung transplantation, this propensity for ectopic lymphoid structure formation, particularly iBALT, may permit earlier and more sustained local initiation and remodeling of humoral immunity within the allograft (22, 23).

Airway instrumentation, antibiotic exposure, and immunosuppressive therapy can alter microbial burden and community composition in the lower airways, thereby promoting persistent low-grade PAMP signaling. Clinical studies have shown that increased microbial burden or dysbiosis in the lower airways of lung transplant recipients is associated with alveolar inflammation and adverse outcomes, including chronic lung allograft dysfunction (CLAD) (24, 25). Therefore, the vulnerability of the lung allograft reflects not only its exposed anatomy and fragile airway barrier, but also the immunologic tension inherent to a mucosal environment under continuous antigenic stimulation, in which immunosuppression must balance control of alloimmunity against preservation of antimicrobial defense. Once this balance is disrupted, the clinical consequence may manifest as infection, rejection, or a combination of both, ultimately contributing to progressive long-term allograft injury (26).

### 1.2 Stage-specific immune activation after lung transplantation and the limitations of immunosuppression

Immune injury after lung transplantation evolves in a stage-dependent manner. The perioperative period is dominated by ischemia–reperfusion injury (IRI) and its clinical manifestation, primary graft dysfunction (PGD), followed by acute cellular rejection (ACR) and AMR. In the longer term, injury may progress to CLAD, which can further present as bronchiolitis obliterans syndrome (BOS) or restrictive allograft syndrome (RAS) (27). This temporal sequence suggests that distinct immune mechanisms predominate at different stages after transplantation.

Among these events, IRI represents a key initiating trigger of perioperative immune activation and can potently induce local innate immune responses. IRI, together with post-transplant infection, promotes the release of damage-associated molecular patterns (DAMPs), which activate resident macrophages and dendritic cells through Toll-like receptor (TLR) signaling and subsequently trigger downstream pro-inflammatory cytokine cascades (28). The resulting inflammatory milieu further recruits and activates alloantigen-specific T lymphocytes, thereby linking innate and adaptive immunity (29).

T cells are the traditional mediators of ACR, where CD8^+^ cytotoxic and CD4^+^ helper subsets proliferate and infiltrate graft tissues. Sustained antigen exposure promotes CD4^+^ T cell differentiation, which in turn facilitates B cell activation (30, 31). Activated B cells may subsequently differentiate into antibody-secreting cells and contribute to humoral alloimmunity, including DSA formation and, in some contexts, autoantibody responses against lung-associated self-antigens (9). Accordingly, standard first-line immunosuppressive regimens are largely centered on T cell inhibition and typically include calcineurin inhibitors (CNIs; such as cyclosporin A [CsA] or tacrolimus [TAC]), antimetabolites such as mycophenolate mofetil, and corticosteroids, although these approaches may not adequately control humoral immune responses (32).

Despite immunosuppression, many recipients exhibit persistently activated pulmonary immunity, including residual effector T cells (33). This chronic alloimmune state contributes to long-term injury and highlights the limitations of current regimens. In addition to driving the canonical innate immune–T-cell inflammatory axis, IRI has also been shown to directly recruit and activate B cells, initiate humoral immune responses, and thereby promote chronic fibrosis and CLAD (34).

When IRI coexists with infection-related inflammatory stimuli, B cell activation may not be fully dependent on T cell help. DAMP/PAMP-driven TLR signaling, complement-associated cues, and survival and differentiation factors such as BAFF and APRIL may collectively form a T-cell-independent (TI) activating axis. When Tfh-cell support is impaired or germinal center (GC) responses are constrained, this axis may still drive B cells toward rapid extrafollicular (EF) differentiation into plasmablasts or antibody-secreting cells (ASCs). The resulting humoral remodeling is characterized by rapid EF effector responses, potentially at the expense of repertoire quality and durability (35–37).

Recent evidence suggests that recipient-derived B cells can rapidly enter the graft after reperfusion and actively participate in the regulation of IRI. Following activation through TLR4–TRIF-associated signaling, these B cells upregulate and secrete monocyte chemokines such as CCL7, promote the recruitment of CCR2^+^ classical monocytes, and further amplify subsequent neutrophil extravasation and tissue inflammation, thereby aggravating early graft injury (38).

On the other hand, antigenic complexes released during tissue injury, together with complement-associated signals, may enhance BCR-mediated antigen recognition (39–41). Synergistic signaling between TLRs and the BCR can lower the activation threshold of B cells and amplify downstream inflammatory transcriptional programs, thereby biasing them toward chemokine production and rapid effector differentiation (42). This mechanism further suggests that immunosuppressive regimens centered primarily on T cell inhibition may not adequately control dysregulated humoral immunity. Taken together, these observations indicate that post-transplant immune injury cannot be fully understood through a T-cell-centered framework alone, and provide a rationale for further examining the diverse roles of B cells in allograft immune regulation and dysfunction.

## 2 B cell function and immune dysregulation

### 2.1 Lineage differentiation of B cells and multipath antibody responses

While T cells have traditionally been the primary focus in transplant immunology, B cells possess diverse and dynamic functions in post-transplant immune regulation. In addition to differentiating into ASCs that mediate humoral immunity, B cells also serve as potent antigen-presenting cells (APCs). Through MHC II–mediated antigen presentation and cytokine secretion (e.g., IL-6, IL-10), they regulate CD4^+^ T cell activation and promote T follicular helper (Tfh) cell development (8, 43, 44). In environments with low antigen availability, memory B cells outperform dendritic cells in recognizing sparse antigenic signals and can rapidly activate memory T cells (45, 46). Accordingly, B cells contribute both to the initiation and amplification of primary immune responses and to rapid recall responses upon repeated antigen exposure. These functions are often shaped by the anatomical site of activation, the response pathway engaged, and the surrounding local microenvironment.

In lung transplantation, these responses show marked compartmentalization. In addition to GC reactions in secondary lymphoid organs, local humoral responses may develop within the allograft through EF pathways, iBALT, or other TLS-like structures, where *in situ* antigen presentation and T–B cell interactions can occur (47).

In clinical follow-up, the most accessible measures are typically peripheral B cells subsets and serum DSAs. Although these indicators are useful for dynamic monitoring, they do not necessarily reflect the immune processes occurring locally within the allograft, including those in bronchoalveolar lavage fluid (BALF) or tissue. This limitation is particularly relevant when effector cells are tissue-resident or locally enriched, in which case blood-based measurements may underestimate the intensity of intrapulmonary immune activity (22). Therefore, data derived from peripheral blood and from BALF or tissue samples should be regarded as complementary sources of evidence rather than direct substitutes for one another (27).

**Figure 1:**
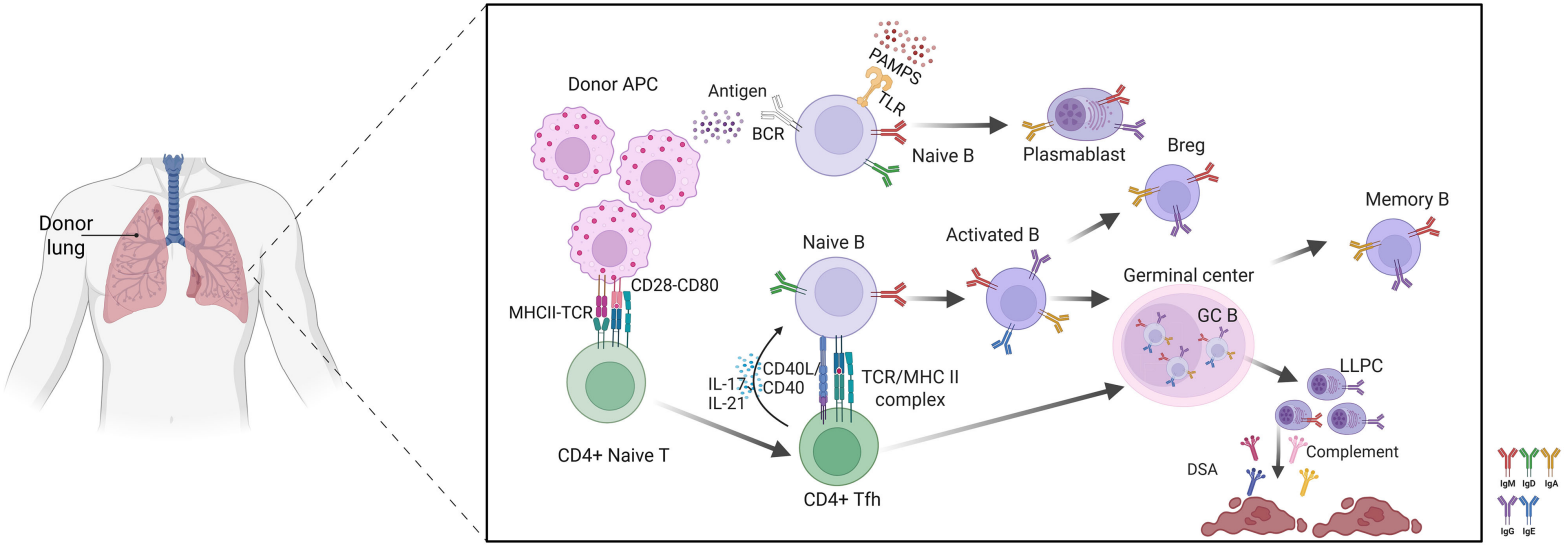
Schematic overview of B cell activation, differentiation, and donor-specific antibody (DSA) production following lung transplantation. Naïve B cells are activated via antigen presentation and TLR signaling, supported by CD4^+^ T cell help, leading to differentiation into plasmablasts, regulatory B cells (Bregs), memory B cells, or germinal center (GC) B cells. GC B cells further give rise to long-lived plasma cells (LLPCs) that secrete DSAs and contribute to complement-mediated graft injury.

B cells development proceeds through well-defined stages governed by B cell receptor (BCR) signaling, from bone marrow-derived Pro B and Pre B cells to transitional and naïve B cells (48). Naïve B cells initiate primary immunity by presenting antigens and activating T cells (49). Upon antigen re-exposure, memory B cells rapidly mount secondary responses, while plasmablasts and plasma cells function as terminal effectors, secreting DSAs that may contribute to AMR. Following antigen stimulation, B cells typically migrate to GC, where they undergo class-switch recombination and affinity maturation. This process is supported by cytokines such as IL-17, which enhances B cell–CD4^+^ T cell interactions and helps maintain GC integrity. Only high-affinity B cells are selected to become memory or long-lived plasma cells, sustaining humoral memory (**Figure 1**).

B cells produce various antibodies relevant to transplantation, including natural antibodies (nAbs), DSAs, and autoantibodies. nAbs, mainly IgM from innate-like B cells, contribute to the clearance of apoptotic cells and the maintenance of immune homeostasis (50). In lung transplantation, nAbs may bind injury-associated neoepitopes during IRI and activate complement, suggesting a role in early innate graft injury rather than in canonical alloantibody-mediated rejection (51, 52).

In contrast to natural antibodies, DSAs arise via GC responses under Tfh guidance (53). Memory B cells can undergo somatic hypermutation (SHM) and class switching and differentiate into plasmablasts or IgG-secreting plasma cells, thereby contributing to DSA formation and antibody-mediated graft injury in lung transplantation (9). In lung transplantation, although serum DSA positivity represents an important immunological clue for AMR, its diagnosis still requires integrated assessment of allograft function, histopathological injury, complement activation, and exclusion of alternative causes such as infection. DSAs target graft endothelial HLA antigens, initiating complement activation and Fc receptor–mediated cytotoxicity, leading to persistent inflammation and graft injury (54). CLAD represents a persistent decline in lung allograft function, including BOS and RAS phenotypes, in which B cell–mediated immunity is one important but not exclusive pathogenic component. Interestingly, some DSA-negative recipients still experience rejection, suggesting that EF responses may contribute (36, 55). EF B cells rapidly differentiate into plasmablasts with limited Tfh input, producing lower-affinity antibodies and generating long-lived plasma cells or durable memory (56). This may explain delayed DSA detection or persistent injury despite negative serology.

Emerging evidence suggests that tissue-resident plasmablasts can arise during alloreactive responses, underscoring the limitation of relying solely on circulating DSA levels to assess B cell activity. A more nuanced understanding of B cell activation routes and differentiation outcomes is essential to improve early diagnosis and guide therapeutic targeting in CLAD (57).

### 2.2 The immunological duality of B cell subsets

B cells play a dual role in transplantation, contributing to both allograft injury and immune tolerance through distinct lineage trajectories and functional subsets (9, 58). On the pro-rejection axis, diverse memory B cell populations—including T-bet^+^CD27^+^CD21^-^ and CD27^-^IgD^-^ double-negative (DN) memory B cells—as well as CD38^+^ plasmablasts, are key drivers of humoral alloimmunity. These subsets can present alloantigens to T cells and differentiate into plasma cells, producing DSAs that activate complement cascades and mediate antibody-driven injury such as AMR and CLAD (46, 59–62). Elevated levels of activated subsets before transplantation have been linked to rejection risk (63).

In CLAD, particularly BOS and RAS innate-like DN memory B cells (CD27^-^ IgD^-^) and tissue-infiltrating CXCR3^+^ ITGB1^+^ B cells have been implicated in local immune remodeling (60, 64). Peripheral B cell dynamics mirror these intragraft events. In BOS and RAS patients, circulating memory B cells increase, while naïve and transitional B cells decline, indicating systemic remodeling (65). In contrast, operationally tolerant recipients exhibit elevated B cell counts enriched in memory and transitional subsets expressing both co-stimulatory (e.g., BANK1) and inhibitory (e.g., FcγRIIB) markers, suggesting a regulatory phenotype that supports long-term graft survival (66).

Temporal fluctuations are also notable: a transient increase in total B cells is often observed at 1 month post-transplant, typically dominated by naïve B cells. In the absence of rejection, plasmablast levels remain low; however, in patients with acute rejection or infection, CD38^+^ plasmablasts expand and contribute to DSA production (67, 68).

**Figure 2:**
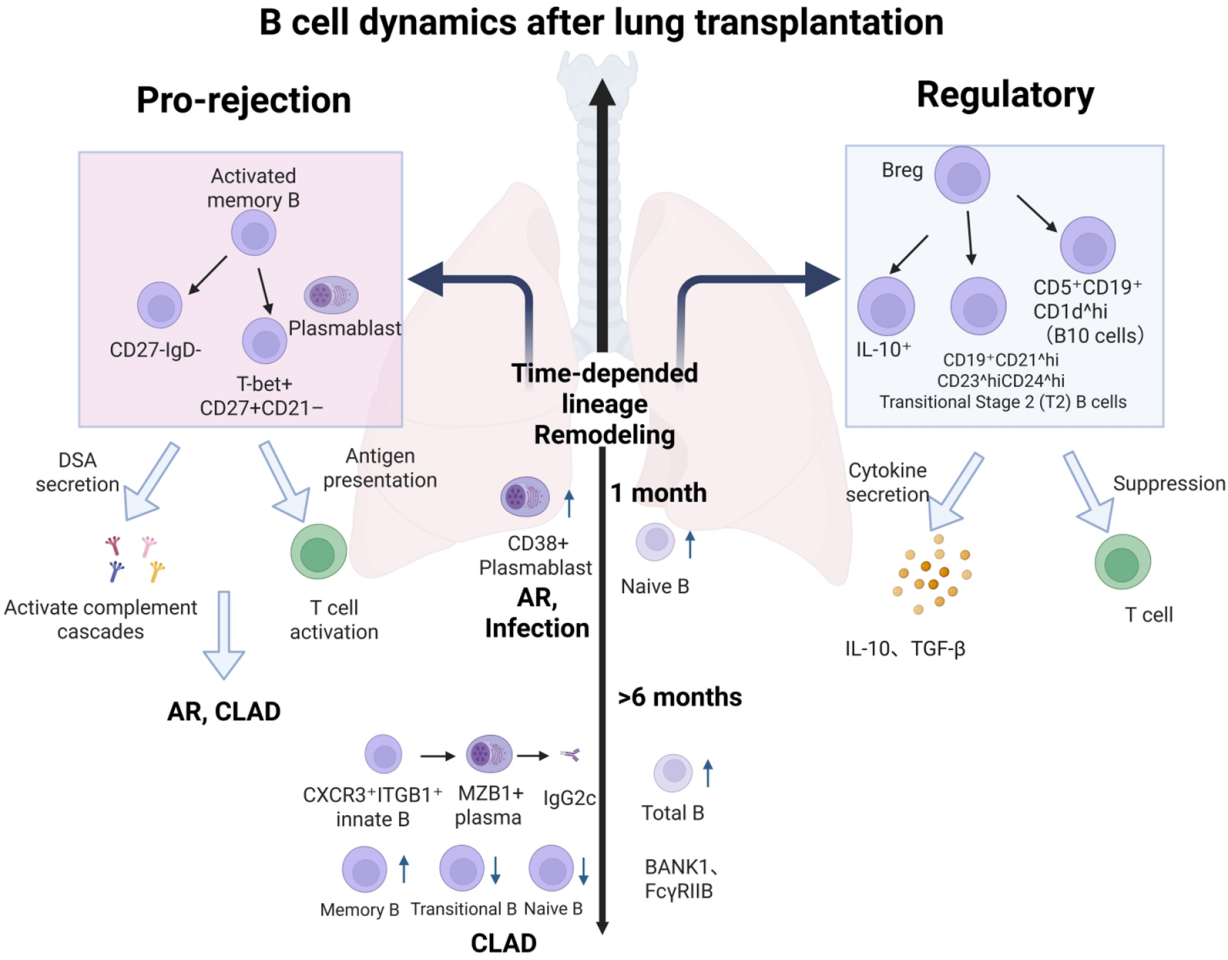
Different B cell subsets exhibit distinct functions and undergo temporal shifts after lung transplantation, contributing to various post-transplant complications.

Balancing this effector activity, regulatory B cells (Bregs) maintain immune homeostasis by producing IL-10 and TGF-β and expressing PD-L1, thereby suppressing T cell activation and promoting tolerance (58, 69). Bregs should be regarded as a heterogeneous functional state rather than a fixed lineage defined by a single surface marker. Reduced Breg frequencies or impaired function have been associated with impaired immune regulation and increased rejection risk. Several phenotypes have been proposed for Bregs, including IL-10^+^ CD5^+^ CD1d^hi^ “B10” cells, CD19^+^CD21^hi^CD23^hi^CD24^hi^ transitional B cells, and CD9^+^ IL-10^+^ subsets. Among these, CD9^+^ B cell signatures have been associated with long-term graft stability after lung transplantation (70). These subset-specific functions and their time-dependent shifts are summarized in **Figure 2**.

These findings underscore that B cell responses in transplantation are temporally dynamic, spatially compartmentalized, and functionally heterogeneous. The balance between pro-inflammatory and regulatory B cell subsets—shaped by inflammatory cues, alloantigen stimulation, and local microenvironments—critically determines graft outcome. Recent lung-transplant-specific evidence linking Pseudomonas aeruginosa infection to intragraft T-bet^+^/CXCR3^+^ B cell expansion and AMR further supports the need for refined B-cell-directed strategies that limit pathogenic humoral responses while preserving regulatory immune functions (71).

### 2.3 Limitations of B cell subset monitoring and the predictive potential of molecular markers

Routine post-transplant immune surveillance primarily relies on DSA detection and quantification of peripheral B cell subsets. While informative, these methods are inherently static and often fail to capture dynamic immune events. Notably, shifts in B cell composition and function often precede DSA emergence or clinical rejection but remain undetected by conventional monitoring. This gap has driven interest in molecular markers that reflect B cell activation, differentiation trajectories, and effector functions. Transcriptomic, proteomic, and epigenetic profiles offer earlier and more precise insights into graft-specific immune remodeling, potentially enabling proactive, individualized immunosuppression.

Pre-transplant detection of IgG4 antibodies has been proposed as a marker of prior, late-stage GC–dependent humoral responses and may predict subsequent IgG3 production and elevated AMR risk (72). However, flow cytometry analyses (e.g., CD27/IgD profiles) have shown limited sensitivity, as demonstrated by van de Berg et al. across multiple post-kidney-transplant timepoints (6 months, 3 and 5 years) (73). This lack of distinction may be attributable to variability in sampling timepoints or to cryopreservation-induced phenotypic alterations, highlighting the need for standardized sample processing protocols in B cell–focused studies (74). Moreover, most longitudinal studies on B cell subsets have been conducted in kidney transplant recipients and are generally limited to later timepoints—typically at 6 months, 2 years, or 5 years post-surgery. These limitations reflect both sample variability (e.g., cryopreservation artifacts) and a broader lack of early-phase (≤ 3 months) data in B cell monitoring.

Additionally, many studies overlook plasmablasts and plasma cells, critical effector subsets in AMR pathogenesis. While total peripheral B cell counts typically decline post-transplant—largely due to loss of naïve B cells—this metric poorly correlates with immune activation. Memory B cells may increase slightly, but the mechanisms behind such shifts remain unclear, influenced by immunosuppression, antigen persistence, or latent viral reactivation. As such, B cell enumeration alone offers limited predictive value. To enhance immune risk stratification, conventional subset analysis must be paired with advanced tools such as BCR repertoire profiling and functional phenotyping. These platforms provide high-resolution data on clonal expansion, activation states, and antibody potential—critical for identifying subclinical immune dysregulation.

Recent studies highlight gene expression signatures linked to B cell activation. During acute rejection, peripheral B cells show downregulation of TCL1A and CD79B, markers of unactivated B cells (75). This shift may indicate differentiation toward antibody-secreting phenotypes. However, similar signatures may arise in unrelated inflammatory settings (e.g., viral infections), raising concerns about specificity. Thus, B cell–based molecular markers hold promise, but require further validation in larger, prospective transplant cohorts, especially during early postoperative periods when intervention may be most impactful.

## 3 Indirect modulation of B cells by immunosuppressants: mechanistic differences between TAC and CsA

### 3.1 Distinct mechanisms of calcineurin inhibitors in B cell regulation: Tfh suppression vs plasma cell targeting

TAC and CsA, the most common calcineurin inhibitors (CNIs) in lung transplantation, are typically administered alongside antimetabolites and corticosteroids (32, 76). Both inhibit T cell activation via the calcineurin–NFAT pathway, reducing IL-2, IL-4, and CD40L expression, thus lowering ACR risk. Although CNIs primarily target T cells, they may also indirectly modulate B cell responses by altering T cell help, humoral activation, and DSA formation. TAC and CsA differ in their downstream effects on B cells, which may influence long-term graft outcomes and immune homeostasis.

Studies in transplant immunology suggest that TAC may suppress B cell activity by inhibiting Tfh cells, thereby reducing plasmablast and memory B cell formation, and lowering immunoglobulin production (10, 77). In kidney transplantation, *de novo* DSA development within the first post-transplant year has been associated with increased CD40L and PD-1 expression on circulating Tfh cells (cTfh), suggesting that CD40L^+^PD-1^+^ cTfh cells may reflect CNI responsiveness and humoral alloimmune risk (78). In lung transplantation, belatacept has been explored as a CNI-sparing strategy in pilot and retrospective studies, but its effects on B cell subsets and DSA kinetics remain insufficiently defined (79, 80).

Lung-transplant-specific evidence indicates that CNI selection has clinical relevance beyond general T cell suppression. An ISHLT registry analysis showed that CsA-based maintenance therapy, compared with tacrolimus immediate-release therapy, was associated with increased CLAD risk and reduced allograft survival (81). Peripheral B cell remodeling has also been reported in lung transplant recipients with post-transplant infection, suggesting that infectious and immunosuppressive contexts may jointly shape B cell subset composition (82). By contrast, evidence linking CNI exposure to B cell transcriptional signatures, including TCL1A-related programs, is derived mainly from kidney transplantation and should be considered indirect evidence requiring lung-specific validation (83–85).

Unlike TAC, CsA may more directly affect antibody-secreting cells (ASCs) by inducing ER stress and activating unfolded protein response pathways, including PERK and ATF6 (86–88). Because plasma cells depend on ER expansion for sustained immunoglobulin production, they may be vulnerable to ER-stress-mediated disruption of protein synthesis and survival. These mechanistic observations provide a rationale for considering plasma-cell stress pathways in AMR, although their therapeutic relevance in lung transplantation remains to be validated (89).

### 3.2 Broader immunosuppressive effects on B cell subsets and tolerance-associated signatures

Immunosuppressive agents may reshape B cell homeostasis beyond their primary effects on T cell activation. In lung transplant recipients, alterations in peripheral CD19^+^CD24^hi^CD38^hi^ regulatory B cell populations have been reported, suggesting that Breg-related immune regulation may be influenced by the post-transplant inflammatory and immunosuppressive milieu (90). By contrast, evidence linking corticosteroids, azathioprine, and CNIs to tolerance-associated B cell genes or specific. B cell subset shifts is derived mainly from kidney transplantation and should be considered indirect evidence (91, 92). Whether these B cell phenotypes are reproducibly associated with DSA development, AMR, CLAD, or infection risk in lung transplantation remains insufficiently defined.

These observations caution against interpreting “tolerance-like” B cell signatures as intrinsic regulatory programs without accounting for immunosuppressive exposure. Functional validation is therefore required before such markers can be applied to risk stratification in lung transplantation.

To enable systematic comparison, **Table 1** summarizes the main B cell subsets affected by commonly used immunosuppressants, along with their detection time points and mechanistic characteristics. This framework distinguishes lung-transplant-specific evidence from indirect observations derived from kidney transplantation or mechanistic studies.

**Table 1:** Effects of common immunosuppressive agents on B cell subsets and clinical relevance in organ transplantation.

Drug class	Drug name	Main affected B cell subsets	Detection time	Evidence source	Clinical interpretation	References
CNI (Calcineurin Inhibitors)	TAC	Tfh-dependent B cell activation and antibody production	One week before surgery/post-transplant immune monitoring	Kidney transplantation	TAC may indirectly suppress B cell activation, plasmablast responses, and antibody production by inhibiting Tfh activation, including reductions in CXCR5^+^ICOS^+^PD-1^+^ Tfh cells and IL-21; its B cell subset effects in lung transplantation require validation	(56)
	TAC	Tfh-supported B cell activation	6-day co-culture ex vivo	Ex vivo mechanistic evidence	Reduces cells co-expressing activation markers CD40L and ICOS, suppresses IL-2 mRNA expression, and indirectly limits B cell activation through impaired T cell help	(10)
	High-dose TAC	TCL1A-expressing B cells	Maintenance immunosuppressive therapy	Kidney transplantation	Tacrolimus-based immunosuppression has been associated with TCL1A-related transcriptional changes in PBMCs	(84, 85)
	CsA	ASC	—	*In vivo* mechanistic evidence	Activates UPR (PERK/ATF6) pathway, induces ER stress response, suppresses persistent antibody secretion	(86–89)
Antimetabol-ites	Azathioprine (AZA)	Transitional, naïve B cells	—	Kidney transplantation	Significantly decreased in number; downregulates drug-resistance-related genes (e.g., CD79b, TCL1A)	(91, 92)
	Mycophenolate mofetil	Transitional B cells	—	Kidney transplantation	Drug concentration–dependent effect, alters B cell subset structure and gene expression related to drug sensitivity	(91)
Costimulation Blockade	Belatacept	Naïve, transitional B cells	*De novo* regimen or conversion after CNI intolerance	Kidney transplantation studies; lung transplantation clinical experience as CNI-sparing therapy	Increased ratio of these subsets, suggests possible promotion of B cell homeostasis maintenance	(80, 115)

## 4 Prospects of BCR repertoire analysis in lung transplantation immunology

### 4.1 Technical basis and clinical potential of BCR immune repertoire sequencing

Each B cell expresses a unique BCR generated by somatic recombination, conferring antigen specificity at the clonal level. Theoretical BCR diversity exceeds 10^12^, supporting a broad immune recognition repertoire (93). Upon alloantigen stimulation, B cells undergo SHM and class-switch recombination (CSR), producing molecular signatures of antigen affinity and isotype switching (94). High-throughput BCR sequencing captures these changes, enabling dynamic analysis of clonal expansion, memory formation, and immune regulation.

BCR repertoire analysis characterizes IGH transcripts by profiling CDR3 sequences, SHM rates, and V gene usage, serving as high-resolution indicators of immune status in transplant recipients (95). Initially developed for infection, autoimmunity, and cancer (96–99), it has shown promise in transplantation. Although direct lung-transplant-specific BCR studies remain limited, evidence from other transplant settings suggests that rejection may be accompanied by intragraft clonal expansion, altered SHM patterns, and EF-like B cell responses under chronic alloimmune stimulation (100, 101).

### 4.2 Interpreting BCR repertoire features through B cell differentiation pathways

After alloantigen recognition, activated B cells may enter either EF or GC differentiation pathways, each leaving distinct molecular imprints in the BCR repertoire. EF responses are typically associated with rapid plasmablast differentiation, limited SHM, and broader antibody reactivity, whereas GC responses are characterized by iterative SHM, affinity selection, and the generation of memory B cells or long-lived plasma cells (36, 102, 103). Thus, the relative contribution of EF and GC responses can help interpret clonal expansion, SHM burden, isotype distribution, and potential pathogenicity in transplant recipients.

EF and GC programs may coexist within a single clonal lineage, which complicates the interpretation of expanded B cell clones. GC-derived clones are generally enriched for SHM and affinity maturation, whereas EF-derived clones may show lower SHM, broader reactivity, and greater plasticity (104, 105). In transplantation, this distinction is relevant because expanded low-SHM clones may reflect recent or ongoing EF activation, whereas highly mutated clones may indicate prior GC experience or memory recall.

Antibody isotypes can also provide clues to humoral immune history. For example, pre-transplant IgG4 has been associated with prior GC activity and later IgG3 production, suggesting a potential link between isotype switching history and AMR risk (94). Integrating isotype distribution, SHM burden, and clonal expansion may therefore improve the interpretation of BCR repertoire data, although these features should be assessed together with DSA status, tissue or BALF findings, infection status, and longitudinal graft function.

### 4.3 Pre-transplant risk assessment and post-transplant dynamic monitoring value

The diversity and clonal evolution of BCR repertoires provide insights into immune status before and after transplantation. Studies in other solid organ transplants have shown that increased pre-transplant BCR diversity, post-transplant diversity contraction, and expansion of specific clones may be associated with rejection-related immune activation (101). Enrichment of selected V genes, such as IGHV3-23, may reflect antigen-driven selection or microbial cross-reactivity, but its transplant-specific interpretation remains context dependent. Newell and Sagoo identified a tolerance-associated B cell gene signature (IGKV4-1, IGLL1, IGKVD-13) in peripheral blood of tolerant kidney transplant recipients, validated in urine by qPCR (106, 107). These findings from non-lung transplant settings support the potential of BCR repertoire analysis as a candidate monitoring tool, but its predictive utility in lung transplantation remains to be established. In lung recipients, such data should be interpreted in conjunction with DSA testing, BALF or tissue findings, infection status, immunosuppressive exposure, and longitudinal lung function.

Evidence from heart and intestinal transplantation further suggests that BCR-related changes may precede or accompany broader immune activation, but these signals are not necessarily rejection-specific (108, 109). Some patients display BCR changes in the absence of rejection, supporting its role in immune surveillance. Specific IGHV enrichment and CDR3 motif changes may serve as immune fingerprints for rejection risk, supporting the development of minimally invasive monitoring tools (110).

### 4.4 Auxiliary value of BCR repertoire analysis in B-cell-directed immunotherapy

B cells are key targets in humoral immunotherapy, and BCR repertoire analysis may help evaluate treatment-related changes in clonal architecture. CD20-targeting rituximab depletes mature B cells, thereby attenuating downstream humoral responses (111, 112). Other B-cell-directed agents, such as CD22-targeting approaches, have been explored in immune-mediated diseases, but their relevance to lung transplantation remains uncertain. Monitoring clonal expansion, diversity, and isotype distribution may provide complementary information on immune modulation (113). However, aberrant clonal expansion should be interpreted cautiously, as it may reflect alloantigen exposure, infection, cytokine dysregulation, or treatment-related immune remodeling.

In cGVHD, recipient stromal cell-derived BAFF and pathogenic donor CD4+ T cell responses jointly contribute to an elevated BAFF/B cells ratio, thereby sustaining aberrant BCR activation (114). BAFF enhances responsiveness by upregulating NOTCH2 and activating SYK, promoting alloantibody production independently of T cell help. These findings highlight the diagnostic and mechanistic value of BCR repertoire dynamics and position the BAFF–BCR axis as a therapeutic target (9, 34, 64, 71). Thus, BCR repertoire analysis may help refine B-cell-directed immunotherapy by identifying treatment-responsive clonal patterns and candidate cytokine–BCR signaling axes, such as BAFF–BCR, that warrant lung-transplant-specific validation.

## 5 Conclusions

Aberrant humoral immunity after lung transplantation is not merely reflected by increased antibody levels, but also by B cell lineage remodeling and adaptive changes within the graft microenvironment, which may contribute to chronic rejection and divergent long-term outcomes. Importantly, the mechanisms of B cell activation, differentiation, and DSA generation in lung transplantation may not fully mirror those in other solid organs, given the lung’s unique mucosal immune environment and potential for local lymphoid organization. These organ-specific features suggest that immune monitoring and immunomodulatory strategies in lung transplantation should not be directly extrapolated from kidney or heart transplantation. Future management will require integrated assessment of B cell subsets, DSA dynamics, local graft immune activity, infection status, and lung-function trajectories to support earlier risk identification and more individualized immunomodulation.
